# A compatible exon-exon junction database for the identification of exon skipping events using tandem mass spectrum data

**DOI:** 10.1186/1471-2105-9-537

**Published:** 2008-12-16

**Authors:** Fan Mo, Xu Hong, Feng Gao, Lin Du, Jun Wang, Gilbert S Omenn, Biaoyang Lin

**Affiliations:** 1 Systems Biology Division, Zhejiang-California Nanosystems Institute (ZCNI) of Zhejiang University, Zhejiang University Huajiachi Campus, 268 Kaixuan Road, Hangzhou 310029, PR China; 2 Department of General Surgery, The Second Affiliated Hospital, ShanXi Medical University, 382 Wuyi Road, Taiyuan 030000, PR China; 3 College of Life Science, Zhejiang University Zijingang Campus, Zijinhua Road, Hangzhou 310058, PR China; 4 Center for Computational Medicine and Biology, National Center for Integrative Biomedical Informatics, University of Michigan, 100 Washtenaw Avenue, Ann Arbor, MI 48109-2218, USA.; 5 Departments of Internal Medicine and Human Genetics, University of Michigan, 100 Washtenaw Avenue, Ann Arbor, MI 48109-2218, USA

## Abstract

**Background:**

Alternative splicing is an important gene regulation mechanism. It is estimated that about 74% of multi-exon human genes have alternative splicing. High throughput tandem (MS/MS) mass spectrometry provides valuable information for rapidly identifying potentially novel alternatively-spliced protein products from experimental datasets. However, the ability to identify alternative splicing events through tandem mass spectrometry depends on the database against which the spectra are searched.

**Results:**

We wrote scripts in perl, Bioperl, mysql and Ensembl API and built a theoretical exon-exon junction protein database to account for all possible combinations of exons for a gene while keeping the frame of translation (i.e., keeping only in-phase exon-exon combinations) from the Ensembl Core Database. Using our liver cancer MS/MS dataset, we identified a total of 488 non-redundant peptides that represent putative exon skipping events.

**Conclusion:**

Our exon-exon junction database provides the scientific community with an efficient means to identify novel alternatively spliced (exon skipping) protein isoforms using mass spectrometry data. This database will be useful in annotating genome structures using rapidly accumulating proteomics data.

## Background

Alternative splicing in eukaryotes is a process that enables a single gene locus to encode for multiple protein products. Johnson et al. used exon-junction arrays to profile more than 10,000 multi-exon human genes in 52 tissues and cell lines and they estimated that about 74% of multi-exon human genes are alternatively spliced [1]. Recently, Pan et al. and Wang et al. used mRNA-Seq (mRNA sequencing using the next-generation sequencing) technology and estimated that transcripts from about 92–95% of multiexon genes undergo alternative splicing [2,3]. Alternative splicing events were traditionally identified by aligning cDNAs to genomic sequences. With the vast number of Expressed Sequence Tags (EST) generated by the EST sequencing projects [4], and more recent development in direct mRNA sequencing (mRNA-Seq) by the next generation sequencing technologies, many alternative splicing events of genes were identified and annotated in the human genome.

With the advances in mass spectrometry (MS) and large-scale generation of MS/MS (tandem MS)-based proteomics data, it has become clear that MS-based peptide sequence data can be mined to identify and validate alternative splicing events of genes. Traditionally, mass spectrometry database searches use databases consisting of known or putatively translated protein sequences, which are biased towards well-known proteins or their common alternatively spliced isoforms that exist in the database. More recently, several methods have been proposed to search for identifying novel alternative splicing variants. Edwards [5] proposed a method using ESTs and a sequence database compression strategy to identify alternatively-spliced peptides existing in the EST database from MS data. However, as most EST sequences were only sequenced once and they contained large number of errors, using the ESTs to search against the mass spectrum data, which is also imperfect, often generate low confidence matches, and it is hard to distinguish bad EST sequences from bad or mis-ionized mass spectra. Alternatively, six-frame translated human genome sequences can be used to search MS spectrum data directly [6,7]. Fermin *et al*. were able to identify 282 novel open reading frames with a false discovery rate threshold of 0.05 from the putative six-frame translation of the human genome [7]. However, the six-frame translation approach [6,7] does not take into account all potential splicing possibilities.

We therefore sought to develop a new strategy to build a database for more efficient identification of novel alternative splicing events using MS data. We designed a theoretical exon-exon junction protein database to account for all possible combination of exons for a gene while keeping the frame of translation (i.e., keeping only in-phase exon-exon combinations). Our database was built from the Ensembl Core Database using scripts we wrote in perl, Bioperl, mysql and Ensembl API. It contains every compatible exon-exon junction protein sequence encoded in the human genome. Using liver cancer MS/MS data we generated in the laboratory as an example, we show that our database is useful in identifying from MS/MS spectra novel alternatively spliced protein isoforms derived from exon skipping.

## Results

### Construction of an exon-exon junction database

We built a putative exon-exon junction translation protein database representing all compatible exon-exon junction sequences using the Ensembl Core Database (homo_sapiens_core_45_36h) from . We took 25 amino acid residues for each exon from those compatible exon combinations or the whole exon if an exon codes for less than 25 amino acids. The reason for doing so is described below. A typical ion-trap mass spectrometer has a window size to detect peptides with molecular weight from 500 to 3000 daltons. A peptide with 25 amino acids would have a molecular weight of about 3000 daltons, which is at the upper range of MS detection. The total number of genes in the database was 15, 536; the total number of exons was 203,180. The average junction sequence per gene is 56. We then excluded 282,991 previously described exon-exon junction sequences if they have been annotated in the Ensembl database, since our purpose is to identify novel splicing isoforms. This reduction in the size of the exon-exon junction database saves MS search time. The final database has 873,024 entries and is about 132 Mb in size.

This program was implemented in Perl. The database and source code can be downloaded from our website at .

### Identification of exon skipping events from mass spectrometry data using the exon-exon junction database

To test the ability of our exon-exon junction database in identifying new splicing events, and to test the hypothesis that novel splicing events could be captured by mass spectrometry as peptides spanning new exon junctions, we performed mass spectrum searches against the putative exon-exon junction database with a liver MS dataset we generated in the laboratory [8]. The dataset contains 321 mzXML files with a total of 1, 327,430 mass spectra, of which 14,583 spectra have PeptideProphet scores > 0.9.

We first used the open source search engine X!Tandem to search against our AS database to identify candidate AS forms. From the 321 mzXML files from liver cancer samples, we found 1063 novel exon skipping peptides, of which 421 peptides (39.6%) were uniquely identified as non-redundant. We searched against a sequence-randomized version of the putative exon-exon junction database as a decoy database using X!Tandem; the FDR is 0.98%.

By combining multiple search engines for proteomics, it is possible to improve the sensitivity in identification of peptides from MS/MS data without sacrificing accuracy as demonstrated by Balgley *et al*. [9]. To increase our chance in identifying alternatively spliced isoforms, we performed additional searches using SEQUEST [10], keeping the same pipeline and the same search parameters as with the X!Tandem search. We identified 238 novel exon skipping peptides, of which 79 peptides (33.2%) were uniquely identified as non-redundant. By searching against the same decoy database as described above, the FDR of the SEQUEST result is 4.99%.

Combining these two searches, we identified a total of 488 non-redundant peptides that represent putative novel splicing events (Additional file 1). Among them, 408 were identified only by X!tandem, 66 were identified only by SEQUEST and 13 were identified by both methods. This is similar to what Balgley *et al*. found that the number of proteins with two or more distinct peptides identified by X!Tandem is about 5 times that identified by SEQUEST [9]. Many putative alternatively spliced peptides were identified multiple times and are listed multiple times in the Additional file 1. These 488 peptides were derived from 395 Ensembl genes as some genes have multiple alternatively spliced peptides.

**Figure 1:**
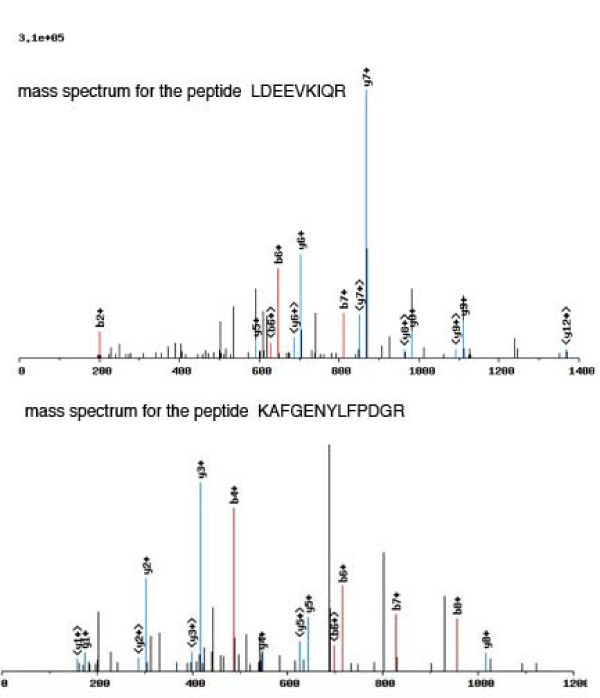
**Top panel, the mass spectrum for the peptide LDEEVKIQR; bottom panel, the mass spectrum for the peptide KAFGENYLFPDGR**. Matched b-ions (e.g. b2+, b6+) are indicated by red, and matched y-ions (e.g. y7+) are indicated by blue. The + indicates that an ion is a singly-charged ion.

**Figure 2:**
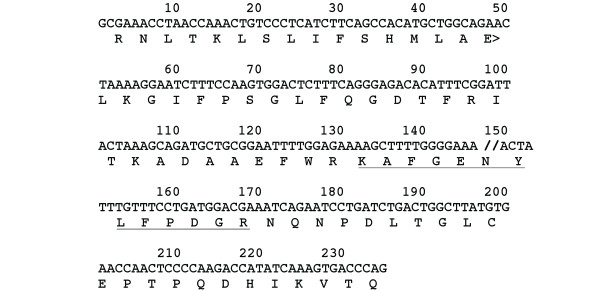
**Illustration of splicing of exons 3 and 7 of the proto-oncogene c-CBL that generates the peptide KAFGENYLFPDGR we identified by MS/MS**. Translated amino acid residues (one letter code) were aligned with the combined exon 3 and 7 DNA sequences. The double forward-slash indicates the exon/exon junction. The underlined amino acid residues indicate the peptide corresponding to the mass spectrum we identified.

Figure 1 (top panel) shows the spectrum of a novel junction peptide LDEEVKIQR, which was not previously identified in either the ECgene or the human non-redundant database. This peptide was observed in both liver cancer and normal datasets. The peptide matched to gene ENSG00000143375, which encodes cingulin. The cingulin gene has 28 exons and is transcribed into a 5142 nucleotide mRNA (NM_020770). The b6 ion for LDEEVK and the b7 ion for LDEEVKI are clearly identified (Figure 1, top panel). The junction position is between amino acid K and I (LDEEVK/IQR, where "/" stands for junction position). It is derived from joining ENSE00000959672 (exon 6) and ENSE00000959682 (exon 21) of the cingulin gene. Cingulin is a protein with modular coiled coil domain, is localized in tight junctions, and may play a role in regulating paracellular permeability [11,12]. The significance and function of this spliced isoform remains to be investigated. The bottom panel of figure 1 shows the spectrum of the peptide KAFGENYLFPDGR. This peptide is derived from splicing of exon ENSE00001240795 (exon 3) and ENSE00001128289 (exon 7) of ENSG00000110395, which encodes for the proto-oncogene c-CBL (E3 ubiquitin-protein ligase CBL). Figure 2 illustrates the splicing event involving exon 3 and 7 of the gene. In our analysis, about 40% of the exon skipping events skipped 7 exons and about 25% of them skipped 14 exons (Additional file 2). Our two examples skip 4 and 15 exons respectively and are typical of the observed skips.

## Discussion

We constructed a putative exon-exon junction database and demonstrated that our database could facilitate the identification of novel alternative splicing variants using high throughput proteomics experimental data. Our approach adds to the methods of identifying alternative splicing forms and is unique. Fermin et al proposed to use the non-redundant database derived from the ECgene database to search for novel putative alternative splicing variants (Fermin D., personal communication). The ECgene database provides annotation for gene structure, function and expression [13]. The ECgene database takes alternative splicing into consideration and is a good starting point for the identification of putative alternatively spliced transcripts. Search against the six-frame translation of the human genome is another approach [6,7]. But the database is more than 300 times the size of the commonly used IPI human protein sequence database, which requires extensive search time as mass spectrum search engines typically require running time linear with size of the sequence database used for searches [5]. In addition, six-frame translation of the human genome did not take into account alternatively spliced exons.

With this exon-exon junction approach, we identified a substantial number of novel alternative splicing junction sequences that were not defined in the public databases, including the comprehensive ECgene database dedicated to identifying human alternative splicing events  and the human non-redundant database (NCBI-nr). We tested our approach using 14,583 spectra from liver cancer cell secretome that have PeptideProphet scores > 0.9. Our MS data is not representative of the whole proteome and the statistics derived from this analysis regarding number of alternative splicing events should not be extrapolated to whole proteome or to other tissues. We merely used this dataset to illustrate the methodology and the idea that mass spectrum data can be used to identify alternatively spliced protein isoforms.

Our liver secretome MS/MS data account for 1, 528 proteins [8]. From these, we identified a total of 488 non-redundant peptides that represent putative novel splicing events (Additional file 1). These 488 peptides are derived from 395 Ensembl genes as some genes have multiple alternatively spliced peptides. The average number of putative splicing events per protein is therefore estimated at 0.32 (488/1528). This is similar to the frequency of 0.33 splice variant per protein from another observation by us, in which we identified 420 splice variant from 1278 proteins from a large dataset of mass spectra derived from plasma proteome of a mouse model of human pancreatic ductal adenocarcinoma [14]. In addition, we compiled a known exon-exon junction database from ECgene and NCBI's non-redundant databases and used it to search the same liver secretome MS/MS data. We identified a total of 1521 known exon-exon junctions based on the same criteria we used to identify novel exon-exon junctions (data not shown). These 1521 known exon-exon junction derived from 1483 genes (1.03 exon-exon junction per gene), which is in contrast with 488 peptides that we identified from 395 Ensembl genes (1.24 exon-exon junction per gene) when we used the novel combinational exon-exon junction database we built. This suggests that there are many novel exon-exon alternative splicing forms that were not captured in the current ECgene or NCBI's nr database.

### Utility

The exon-exon junction database is useful in finding alternative splicing events from high throughput proteomics data generated from modern mass spectrometers. One can simply use this AS junction database instead of the regular protein database in MS database searches, while keeping the search engine and the search parameters the same as searching regular protein databases. The output is a list of hits. One can further process this list using statistics developed for processing MS search results, such as PeptideProphet, to retain only highly confident hits.

The caveat is that our database is based on annotations from ENSEMBL (homo_sapiens_core_45_36h). Our approach is limited to identifying novel splicing forms derived from combinations of known exons. It will not allow the identification of splicing involved in unidentified or un-annotated exons or genes in the human genome. When new ENSEMBL build and new annotations (e.g. new exons) are available, the database should be updated to include new information. We will provide such an updated database when there is a significant new build of the ENSEMBL database.

### Availability

The database can be downloaded from our website at .

## Methods

### Construction of putative Exon-Exon junction translation protein database

Ensembl Core Database (homo_sapiens_core_45_36h) was downloaded from the Ensembl website  into a local MySQL database. We built the putative exon-exon junction translation protein database, taking 25 amino acids from the end of an exon and 25 amino acids from the beginning of the next exon. However, as not all exon-exon combinations from a given gene are compatible, we need to consider combinations of exons that are in-phase. A phase indicates the position within a codon where an exon ends or starts. Phase 0 indicates that an exon ends and starts between two codons; phase 1 indicates that an exon ends and starts between the first and second base of a codon; and phase 2 indicates that an exon ends and starts between the second and third bases of a codon (Additional file 3). Phase -1 (minus 1) indicates that the exon is a non-coding exon (e.g., 5' UTR). We only included combinations of exons that are in phase, which means that the end phase of the first exon must match in frame with the start phase of the next exon, thereby not creating any frame-shift after translation.

### SEQUEST or X!Tandem searches

**Figure 3:**
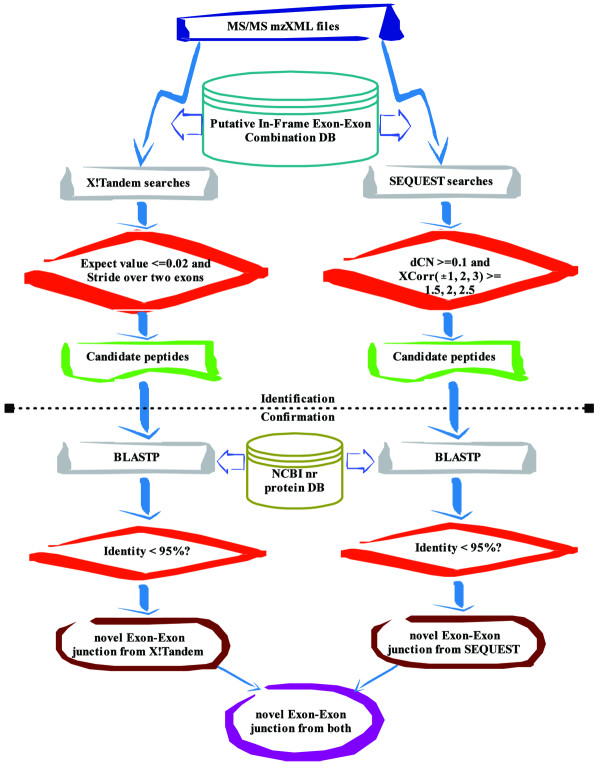
**Flowchart of our pipeline for identifying exon skipping forms using MS/MS data**.

We used two liver mass spectrometry datasets to perform X!Tandem and SEQUEST searches against the constructed putative exon-exon junction protein database. The two datasets (one is cancer, the other is normal) were generated using multiple dimension liquid chromatography (MDLC) coupled to a LTQ-Orbitrap (Thermo Scientific Inc.) mass spectrometer. We converted all RAW mass spectra to mzXML files (160 for liver cancer tissues and 161 for normal adjacent tissues) by ReAdw , then analyzed them using the X!Tandem open source protein identification program [15] or the TurboSEQUEST (Bioworks version 3.2, Termo Electron)[16] (Figure 3). Searching parameters for X!Tandem and SEQUEST were set the same. Searches were performed using a fragment monoisotopic mass error tolerance of 400 ppm and parent monoisotopic mass tolerance of +/- 10 ppm. In the search parameters, we included one static post-translational modification for cysteine (+57.022 daltons) and four optional post-translational modifications (+16 for methionine and + 80 daltons for serine, threonine and tyrosine). Trypsin was used as proteolytic cleavage enzyme, and one missed cleavage site was allowed.

For the X!Tandem search results, the filtering criterion was set at an expect value <= 0.02. For the results from the SEQUEST search algorithm, the filtering criteria for accepting a peptide were set at dCN > 0.1 and X-correlation scores for charge +1, +2, and +3 ions greater than 1.5, 2.0, 2.5, respectively [17] (see Figure 3). We defined a candidate junction peptide as one striding over two exons where there are >= 2 amino acids in each exon matched to a spectrum.

All searches were performed in a Linux cluster based on an MPI (Massive Passing Interface) environment [18]. In order to calculate the false discovery rate (FDR) of the X!Tandem/SEQUEST results, a sequence-randomized version of the putative exon-exon junction database was used as a decoy database to combine with the original database and searched using the same parameters for X!Tandem/SEQUEST. This randomized database was built by replacing each real database entry with a random sequence of the same length but with the average AA composition for the entire database. The perl script used to randomize the database entries was downloaded from . We chose the random option of the perl script (i.e. decoy.pl [--random] input.fasta [output.fasta]), for which the output entries are random sequences with the same average amino acid composition as the input database. The false discovery rate (FDR) was calculated by Ndecoy/Ncombined, where Ndecoy (namely FP, false positives) is number of matches to the entries from decoy database and Ncombine (namely FP+TP, false positives + true positives) is number of matches to the combined database. The FDR controls the expected proportion of false positives under certain threshold for large-scale proteomics data searches [19-21].

### Selection of high-confidence putative exon-exon junction peptides

We wrote perl scripts to parse XML output files of X!Tandem analysis and EXCEL output files of SEQUEST analysis. These output spectra were subdivided into 2 classes: (a) spectra that only match to one exon; (b) spectra that cover junction sequences (striding over two exons where there are >= 2 amino acids in each exon) of two exons. All candidate peptides which belong to class b were aggregated into a FASTA format file for further BLAST analysis.

### Removing known splicing forms by searching against the human non-redundant database

Candidate exon-exon junction peptides were searched against both the ECgene non-redundant database [22] and Human non-redundant database , using BLASTP and the PAM30 matrix [7,23] while turning off the gapped alignment. Only those peptides that have less than 95% identity to or that have no hit in the ECgene non-redundant database or the NCBI non-redundant (nr) database were considered to be novel alternative splicing variants.

## Authors' contributions

BL, MF and HX conceived the research. MF, HX, LD, FG, and JW conducted the research. GSO helped to conduct the research; GSO helped to write the manuscript.

## Supplementary Material

Additional file 1 **Putative novel splicing peptides identified by our approach**. Click here for file

Additional file 2 **A bar chart showing the statistics of the number of skipped exons for the exon-skipping events we identified.**. Click here for file

Additional file 3 **Illustration of the exon phases**. Click here for file
