# Supplementary figures and images for: A compatible exon-exon junction database for the identification of exon skipping events using tandem mass spectrum data
Source: BMC Bioinformatics. 2008 Dec 16;9:537. doi: 10.1186/1471-2105-9-537 (PMC2636810; doi:10.1186/1471-2105-9-537)

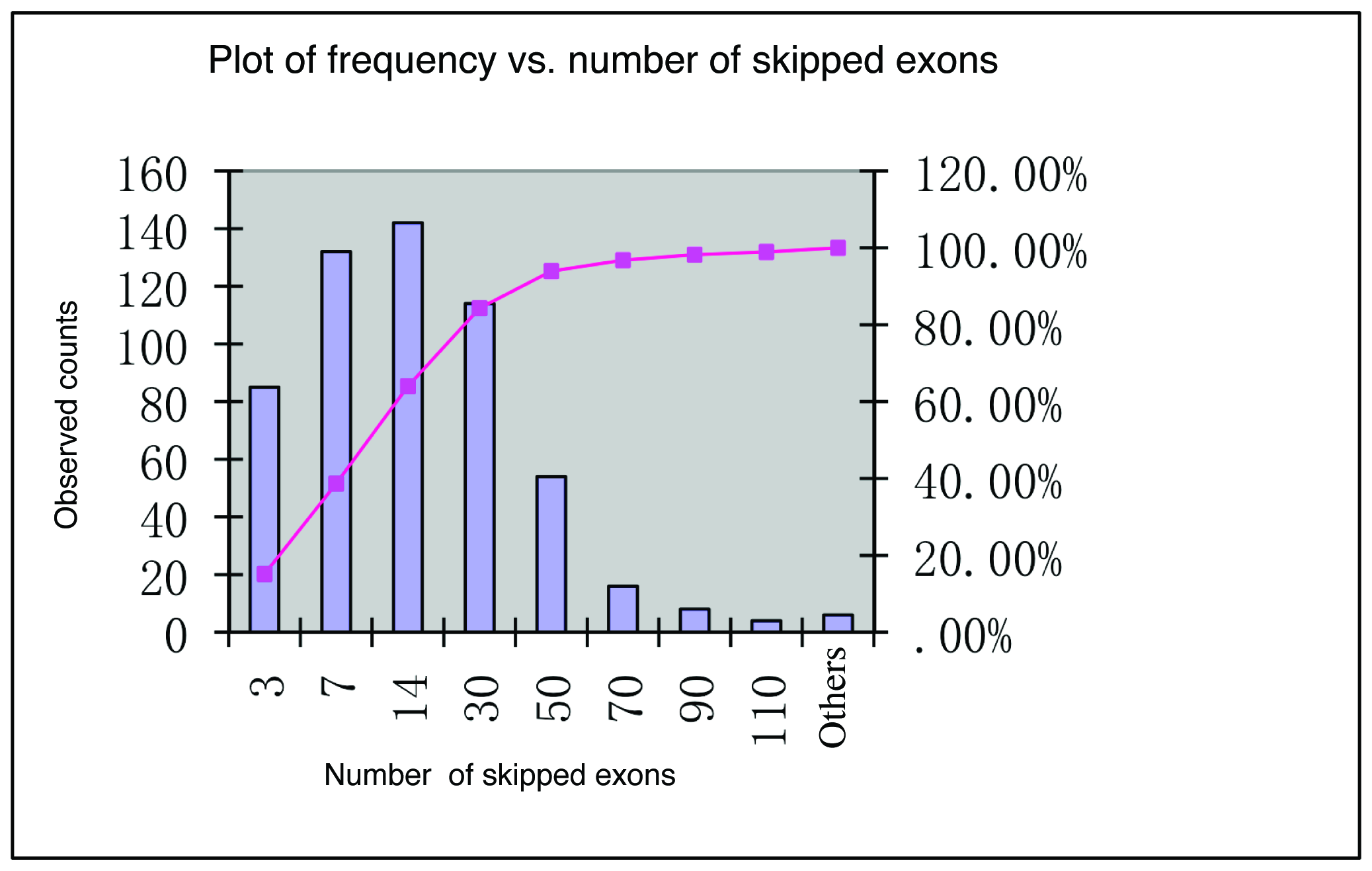

Supplement: Additional file 2 — A bar chart showing the statistics of the number of skipped exons for the exon-skipping events we identified.. [file 1471-2105-9-537-S2.tiff]
